# Forecasting urban air quality in Paris using ensemble machine learning: A scalable framework for environmental management

**DOI:** 10.1371/journal.pone.0336897

**Published:** 2025-11-20

**Authors:** Somia A. Asklany, Doaa Mohammed, Ismail K. Youssef, Majed Nawaz, Wajdan Al Malwi

**Affiliations:** 1 Department of Computer Science, College of Science, Northern Border University, Arar, Saudi Arabia; 2 Department of Mathematics, Faculty of Science, Islamic University of Madinah, Madinah, Saudi Arabia; 3 Department of Informatics and Computer Systems, College of Computer Science, King Khalid University, Abha, Saudi Arabia; Euro-Mediterranean Center for Climate Change: Fondazione Centro Euro-Mediterraneo sui Cambiamenti Climatici, ITALY

## Abstract

Urban air pollution poses a significant threat to public health and urban sustainability in megacities like Paris. We cast forecasting as a short-term, next-hour prediction task for PM2.5, NO, and CO, using hourly meteorology and recent pollutant history as inputs. We develop a data-driven framework based on hyperparameter-tuned ensembles (Random Forest, Gradient Boosting, and a Stacked Ensemble) and benchmark against a Long Short-Term Memory (LSTM) model, alongside persistence baselines. All evaluation metrics (RMSE/MAE) are reported in physical units (µg/m³) with R² unitless. Results show that tree ensembles deliver the lowest errors for PM2.5 and CO, while LSTM is competitive for NO; stacking offers gains when base-model errors are complementary but does not universally dominate. The framework is designed for real-time deployment and integration into smart city pipelines, supporting proactive air quality management. By providing accurate, unit-consistent short-term forecasts, this study informs urban planning, risk mitigation, and public-health protection.

## 1. Introduction

Air pollution is a significant threat to global public health and urban sustainability, contributing to an estimated 7 million premature deaths worldwide each year, according to the World Health Organization. In densely populated metropolitan areas like Paris, the impacts of air pollution are particularly acute. The city’s intricate web of vehicular traffic, industrial zones, residential heating systems, and seasonal meteorological patterns interacts in complex ways to exacerbate the levels of harmful pollutants. These include fine particulate matter (PM2.5 and PM10), nitrogen oxides (NO and NO₂), and carbon monoxide (CO), all of which are associated with a range of health outcomes, from chronic respiratory illnesses and cardiovascular disease to cognitive decline and premature mortality [1].

Paris has taken several legislative and infrastructural steps to mitigate these challenges, such as implementing the Paris Climate Plan, enforcing low-emission zones, and restricting vehicular access in certain districts during pollution peaks [2]. While these measures are impactful, their effectiveness hinges on access to accurate, high-resolution air quality forecasts that enable city planners and public health authorities to act proactively. Yet, traditional air quality models, including deterministic chemical transport models and regression-based statistical approaches, often fall short in dynamically capturing the nonlinear interactions between environmental, anthropogenic, and meteorological variables. In response, data-driven methods such as machine learning (ML) have emerged as promising alternatives [3,4]. ML models, particularly ensemble learning techniques, can accommodate high-dimensional, complex, and noisy datasets without rigid assumptions. However, deploying such models for urban air quality forecasting requires careful calibration, feature engineering, and validation to ensure both interpretability and operational reliability. Moreover, despite their technical merit, ML models must be contextualized within the broader environmental and policy landscape to ensure scientific rigor and real-world applicability [5].

This study addresses this intersection by presenting a scientifically grounded, hyperparameter-tuned ensemble learning framework for air pollution forecasting, using Paris as a case study. We combine Random Forest, Gradient Boosting, and a Stacked Ensemble model (with LightGBM as the meta-learner), trained on hourly air quality and meteorological data from 2023 [6,7]. The framework is benchmarked against a Long Short-Term Memory (LSTM) deep learning model to assess accuracy across pollutants with varying temporal dynamics. Beyond technical performance, we explore the implications of model deployment for real-time environmental intelligence, urban sustainability planning, and public health policy [8,9]. By embedding predictive models within the complex realities of a megacity, this research aims to provide a replicable and scalable solution for cities seeking to balance growth, resilience, and environmental health.

**Fig 1:**
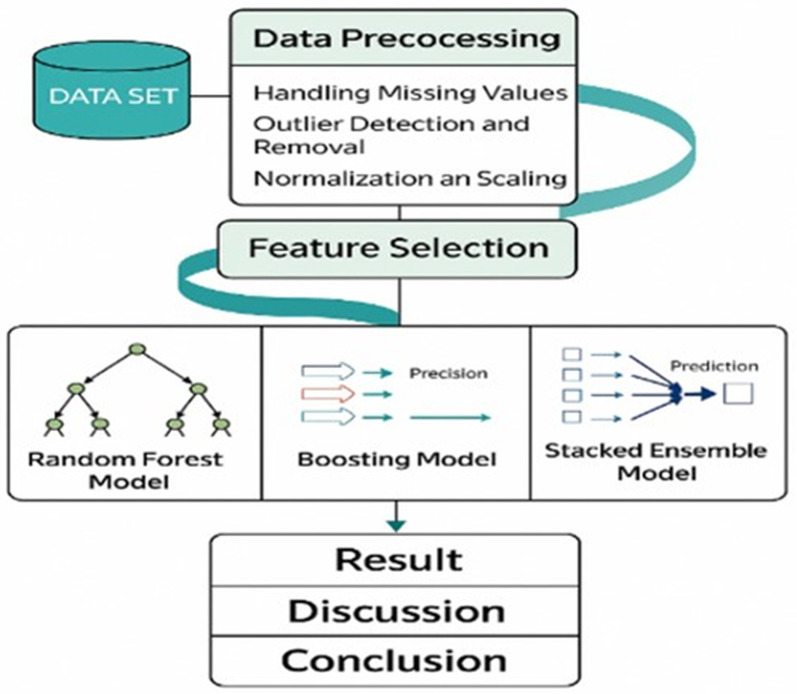
The workflow of the submitted proposal.

In this study, we formulate air quality forecasting in Paris as a short-term, next-hour prediction problem, where the task is to predict concentrations of three key pollutants (PM2.5, NO, and CO) using hourly meteorological variables (temperature, wind speed, sea-level pressure, and visibility) and recent pollutant observations as input features. The workflow of this paper is summarized in Fig 1.

## 2. Related work

Air pollution forecasting has been an active area of research due to its significant impact on public health, environmental sustainability, and urban planning. Traditional approaches, including statistical regression and atmospheric dispersion models, have been widely used but often struggle to capture the complex and dynamic nature of air pollution. With advancements in machine learning (ML) and artificial intelligence (AI), researchers have explored various data-driven models to improve forecasting accuracy.

### 2.1. Traditional air pollution prediction models

Early models for air pollution forecasting relied on linear regression, autoregressive integrated moving average (ARIMA), and Gaussian dispersion models [10]. These approaches required strong domain knowledge and assumptions about the relationships between meteorological variables and pollutant concentrations. However, their primary limitation is the inability to capture non-linear interactions between factors such as temperature, humidity, wind speed, and pollutant emissions [11].

### 2.2. Machine learning-based approaches

The emergence of machine learning has transformed air pollution forecasting by enabling data-driven predictions that do not require explicit assumptions about the underlying relationships. Several studies have applied ML techniques such as Artificial Neural Networks (ANNs), Support Vector Machines (SVMs), and Decision Trees (DTs) to model pollutant concentrations [12]. While these models have shown promising results, they often suffer from overfitting or limited interpretability when dealing with large and complex datasets.

AI and ML techniques have gained traction for their ability to extract complex patterns from large datasets. Artificial Neural Networks (ANNs), in particular, have been widely adopted for forecasting PM2.5 and PM10 levels, demonstrating strong performance across various regions, including Chongqing, China [13]. Notably, the study by Guo et al. (2023) compared 13 ANN training algorithms and found the Bayesian Regularization and Levenberg–Marquardt algorithms for achieving the highest accuracy, with R^2^ values exceeding 0.97 and low RMSE/MAE values for both PM2.5 and PM10. Their results underscore the capability of well-optimized ANN models to achieve high generalizability in real-time pollution forecasting.

ML techniques have shown great promise in capturing the complex interactions among atmospheric pollutants. A recent study focused on Tehran Megacity, employing various ML models, including decision tree, random forest, and gradient boosting, to investigate relationships between PM2.5, NO₂, and CO concentrations [14]. Their analysis highlighted the superior performance of tree-based ensemble models, particularly gradient boosting, in modeling inter-pollutant dynamics under different seasonal and meteorological conditions. Moreover, the study emphasized the importance of feature selection and hyperparameter tuning in improving model interpretability and predictive accuracy. These findings align with the current trend of utilizing ensemble learning to address the nonlinear, high-dimensional nature of air pollution data, reinforcing the potential of optimized ML frameworks in urban environmental management.

Among machine learning (ML) techniques, ensemble learning methods, such as Random Forest (RF) and Gradient Boosting Machines (GBMs), have gained significant attention. These models combine multiple weak learners to create a strong predictive model, enhancing accuracy and generalization. Studies have demonstrated that RF and Boosting outperform standalone models, such as SVMs and ANNs, in air pollution forecasting due to their ability to handle high-dimensional data and complex feature interactions [15,16].

### 2.3. Research gap and contributions

Despite significant advancements in ML-based air pollution forecasting, several challenges remain:

Lack of comparative analysis between different ensemble methods on real-world datasets [17].

Limited use of hyperparameter optimization to fine-tune ML models for air pollution prediction [18]. Few studies on Stacked Ensemble Models that combine RF, Boosting, and other techniques to improve accuracy [19].

This study addresses these gaps by:

- Comparing the performance of Random Forest, Boosting, and Stacked Ensemble models on air pollution forecasting.
- Applying hyperparameter optimization to enhance predictive accuracy.
- Evaluating multi-pollutant forecasting (PM2.5, NO, and CO) in Paris to provide a comprehensive analysis.

The findings contribute to the development of highly accurate, AI-driven pollution forecasting systems, providing policymakers and environmental agencies with valuable insights to mitigate air pollution risks.

## 3. Methodology

This section outlines the methodology employed for air pollution forecasting in the Paris Mega City, focusing on data acquisition, preprocessing, feature selection, machine learning models, and performance evaluation. The proposed approach leverages Random Forest (RF), Boosting, and Stacked Ensemble Models, optimized through hyperparameter tuning to enhance predictive accuracy.

### 3.1. Data acquisition and description

One of Europe’s most densely populated cities, Paris faces persistent air pollution from traffic, heating, and industrial activities. Seasonal factors, especially in winter, intensify PM2.5, PM10, NO₂, NO, and CO levels. The city has implemented mitigation policies, including low-emission zones and traffic restrictions.

This study utilizes hourly air quality and meteorological data for 2023, collected from official monitoring stations in Paris (Latitude: 48.8606342, Longitude: 2.3468978), and supplemented with global datasets. Air pollutant measurements were sourced from OpenAQ, an open-source platform aggregating air quality data worldwide. Meteorological data, including temperature, pressure, and humidity, were obtained from the National Oceanic and Atmospheric Administration (NOAA) levels.

To support the development and evaluation of machine learning models, the dataset was partitioned into 80% for training and 20% for testing. This division facilitates robust assessment of forecasting models under real-world variability.

The data set enables a data-driven analysis of pollution patterns, summarized in Table 1. This allows for the identification of seasonal trends and supports the development of accurate predictive models for air quality forecasting. Preprocessing steps included exploratory data analysis, handling missing values, normalization, and feature engineering to improve the predictive power of the machine learning algorithms.

**Table 1:** Summary of monthly air quality and weather parameters in Paris (2023).

Parameter	Min Value	Max Value	Average Std Dev	Notable Trend
**Temperature**	6.49 (Jan)	22.09 (Jun)	3.96	Rises to a peak in summer
**CO**	0.14 (Jul)	0.32 (Feb)	0.08	Higher in winter
**PM10**	12.45 (Jul)	27.00 (Feb)	8.32	Peaks in winter, lower in summer
**PM2.5**	6.73 (Aug)	18.44 (Feb)	6.71	Winter peak, summer low
**NO**	1.60 (Aug)	15.76 (Feb)	9.4	High winter spikes
**NO**_**2**_	12.82 (Jul)	33.81 (Feb)	13.1	Sharp winter peaks
**Wind Speed**	2.33 (Sep)	4.04 (Mar)	1.36	Relatively stable
**Visibility**	1610.15 (Dec)	2231.99 (Oct)	2411.09	Higher in autumn
**Sea Level Pressure**	1009.12 (Nov)	1029.06 (Feb)	8.17	Peaks in February

**Fig 2:**
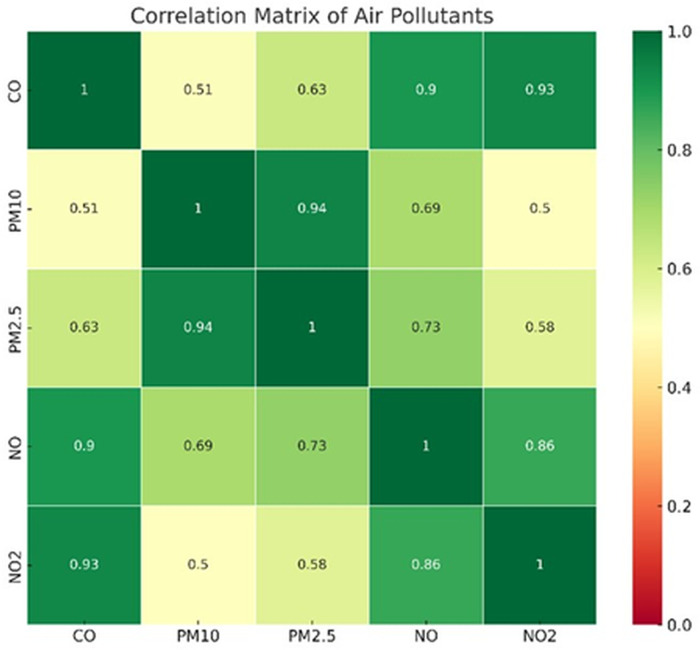
Heat map correlation of different Air pollutant parameters.

Fig 2 explores relationships among the variables in a heat map.

### 3.2. Data preprocessing

Raw environmental data often contains missing values, noise, and outliers, which may degrade model performance in Table 2.

**Table 2:** Summarization of outlier and missing values in the dataset.

Pollutant	CO	PM10	PM2.5	NO	NO_2_
**Total Outliers**	161	1230	15	6	14
**Total Missing**	161	151	159	159	208

The following preprocessing steps were applied:

#### 3.2.1. Handling missing values.

Any missing values were replaced using mean imputation for time-series gaps, and linear interpolation was used to ensure continuity.

#### 3.2.2. Outlier detection and removal.

Outliers were detected using the interquartile range (IQR) and replaced with median values to prevent extreme variations from skewing predictions. Outliers were flagged using the Tukey 1.5 × IQR rule per pollutant and hour; to preserve legitimate peaks, we replaced flagged values with the within-window median rather than deleting them. Across 2023, as shown in Table 2.

#### 3.2.3. Normalization and scaling.

To improve model convergence, all variables were normalized using Min-Max normalization to ensure all features are located within the [0, 1] range.

The transformation equation used was:


$$x^{\prime }=\frac{X-Min(X)}{Max(X)-Min(X)}$$ (1)

Imputation statistics were estimated on the training folds only and applied unchanged to the corresponding validation and test splits to prevent information leakage. We retained mean imputation for transparency and reproducibility because cross-validated RMSE/MAE changed negligibly under a simple time-aware alternative. A small sensitivity check using a time-aware stratified mean imputer produced similar RMSE/MAE and did not change model ranking. As a robustness check, we reran the training with a time-aware stratified mean imputer; the RMSE/MAE and model ranking remained unchanged within rounding, indicating that the imputation scheme does not drive our conclusions.

### 3.3. Feature selection

To avoid information leakage, feature selection was performed strictly within the training data. For each training fold in a blocked time-series cross-validation (CV), we trained a 500-tree Random Forest on the training fold only and computed out-of-bag (OOB) predictor importance. Features were then ranked by importance, and we retained the top 50% (or the proportion selected by inner CV). The selected features from each fold were applied to the corresponding validation split (and, in the final fit, to the held-out Test set) without re-estimating importance on non-training data. After hyperparameter tuning, the models were refitted on the combined training and validation data using the training-derived feature subset and evaluated once on the untouched Test set.

### 3.4. Machine learning models

Three ensemble learning approaches were employed to predict air pollution levels, with their implementation and evaluation discussed in detail in the following sections.

### 3.5. Random forest (RF) model

The Random Forest (RF) model is a robust ensemble learning technique that constructs multiple decision trees and combines their predictions to enhance accuracy and mitigate overfitting. In this study, the RF model was trained using 800 decision trees, with optimized hyperparameters to improve performance. Out-of-bag (OOB) validation was employed to evaluate the model’s effectiveness within the ensemble learning framework.

#### 3.5.1. Optimized hyperparameter elements.

Hyperparameter tuning is vital in enhancing model performance by balancing bias and variance. Key parameters were fine-tuned for the Random Forest model to improve accuracy and generalization.

1. Fine-Tuned Hyperparameters, adjusted to optimize model performance.
2. Minimum Leaf Size (MLS): This controls tree complexity by setting the minimum number of observations required in a leaf node. A node is split only if it contains at least twice the MLS value, ensuring sufficient data in child nodes.

Let ${\;N}_{t}$ be the number of observations in a given node t, and let Minimum Leaf Size be denoted as MLS. The node can only be split further if:


$${\;N}_{t}\geq \text{2}\times MLS$$ (2)

#### 3.5.2. Optimized hyperparameter details.

We optimized model hyperparameters using Bayesian optimization on the training data only. Each candidate configuration was evaluated via five-fold cross-validation within the training set, minimizing cross-validated regression loss (RMSE). The test set remained untouched until the final evaluation. For reproducibility, the random seed was set to 2023.

**Random Forest (bagging) search space (tuned).**
  - NumLearningCycles (trees), optimized
  - MinLeafSize, optimized
  - NumVariablesToSample (features per split), optimized *(tuned).*
**Random Forest final tuned values used (p = 4).**
  - NumLearningCycles = **800**
  - MinLeafSize = **8**
  - NumVariablesToSample = **2**
**Gradient Boosting (LSBoost) search space (tuned).**
  - NumLearningCycles optimized
  - MinLeafSize optimized
  - *(LearnRate was fixed and not tuned in this study.)*
**Gradient Boosting final values used.**
  - NumLearningCycles = **600**
  - MinLeafSize = **8**
  - LearnRate = **0.1**
Model selection and refit

The lowest mean CV loss chose the best hyperparameters (per pollutant/model); models were refit on the training data and evaluated once on the held-out test set. Final tuned values are reported in Table 3.

**Table 3:** Hyperparameter optimization setup and final values.

Model	Optimizer	Acquisition	CV scheme	Objective (CV loss)	Seed	Tuned hyperparameters	Final values used
**Random Forest (bagging)**	Bayesian optimization	expected-improvement-plus	5-fold CV, train-only	RMSE (minimize)	2023	NumLearningCycles, MinLeafSize, NumVariablesToSample	NumLearningCycles = 800; MinLeafSize = 8; NumVariablesToSample = 2
**Gradient Boosting (LSBoost)**	Bayesian optimization	expected-improvement-plus	5-fold CV, train-only	RMSE (minimize)	2023	NumLearningCycles, MinLeafSize (LearnRate fixed)	NumLearningCycles = 600; MinLeafSize = 8; LearnRate = 0.1 (fixed)
**Stacked Ensemble (meta)**	(optional) Bayesian optimization	(optional) expected-improvement-plus	5-fold CV on OOF meta-features	RMSE (minimize)	2023	Meta-learner params (e.g., LSBoost or Ridge)	Meta-learner = LSBoost; NumLearningCycles = 600; MinLeafSize = 8; LearnRate = 0.1 (fixed); OOF-trained

**Stacked ensemble (leakage-safe).** Stacking used **out-of-fold (OOF)** predictions from the tuned Random Forest and Gradient Boosting models under the same 5-fold CV to train the meta-learner, preventing leakage. Final test predictions were obtained by refitting base models on the whole training set and passing their test predictions to the OOF-trained meta-learner.

#### 3.5.3. Feature subset selection in ensemble learning.

In this study, the number of variables sampled at each split was tuned by Bayesian optimization rather than fixed to a default.

#### 3.5.4. Out-of-Bag (OOB) validation in ensemble learning.

Out-of-Bag (OOB) Validation: Used as an internal cross-validation method to estimate model performance without a separate validation set, improving efficiency and robustness of observations.

### 3.6. Boosting model

Boosting is a powerful ensemble learning technique that combines multiple weak learners (typically decision trees) to create a strong predictive model. Unlike bagging methods (e.g., Random Forest), where models are trained independently, boosting models are trained sequentially, with each new model focusing on correcting the errors of its predecessors. The updated model at each iteration is given by:


$$F_{m}(x)=F_{m-\text{1}}(x)+\alpha_{m}h_{m}(x)$$ (3)

Where $\alpha_{m}\;$ is the learning rate, and $h_{m}(x)$ is the weak learner trained on residual errors.

The model minimizes the loss function  L(y, F(x)) by approximating its gradient:


$$h_{m}(x)=-\nabla FL(y,F_{m-\text{1}}(x)$$ (4)

Gradient Boosting iteratively adds weak learners, each fit to the residuals of the current model, so the ensemble focuses on hard-to-predict observations and reduces squared error.

Model specification:

- Gradient Boosting Machine (GBM) with 600 boosting iterations (final selected value).
- Hyperparameters: MinLeafSize tuned via CV; Learn Rate fixed at 0.1.

### 3.7. Stacked ensemble model

Stacked Ensemble Learning (Stacking) is an advanced ensemble learning technique that combines multiple base models (weak learners) to create a stronger, more accurate predictive model. Unlike Bagging (Random Forest) or Boosting (Gradient Boosting, XGBoost), where models are trained independently or sequentially, Stacking uses a hierarchical approach to learn from multiple models and improve overall performance. The stacking is done through two levels, as explained in the next section.

Level 1: Base Models (Weak Learners)
  - A set of diverse machine learning models (e.g., Random Forest, Gradient Boosting, Support Vector Machines, and Neural Networks) is trained on the same dataset.
  - These models make independent predictions.
Level 2: Meta-learner (blender model)
  - The predictions from the base models are used as input features for a meta-learner (also called the blender model).
  - The meta-learner learns how to combine the base model predictions to make the final decision.

In this study, two meta-learners were evaluated in the stacking process: Light Gradient Boosting Machine (LightGBM) and Ridge Regression. These were chosen based on both their complementary strengths and evidence [20].

- LightGBM, is known for its efficiency and high performance in handling large-scale, structured datasets, especially when nonlinear interactions are present.
- Ridge Regression, on the other hand, is a robust linear model that performs well when multicollinearity exists among input features, such as predictions from similar base learners (RF and Boosting).

#### 3.7.1. Meta-learner selection justification.

Preliminary experiments were conducted to evaluate the performance of two candidate meta-learners: Light Gradient Boosting Machine (LightGBM) and Ridge Regression. These were selected for their complementary characteristics LightGBM excels in handling non-linear patterns and large-scale data, while Ridge Regression provides interpretability and robustness to multicollinearity. Table 4 summarizes the performance comparison of both meta-learners across three key pollutants using the same base models (Random Forest and Boosting). Results indicate that LightGBM consistently achieved lower RMSE and higher R² values, especially for NO and CO, albeit with marginal improvements in some cases. Ridge Regression offered faster training time and better explainability, but lagged slightly in predictive accuracy.

**Table 4:** Performance comparison of meta-learners in stacking (validation set).

Pollutant	Metric	Ridge Regression	LightGBM
**NO**	RMSE (µg/m³)	2.722	2.116
**NO**	R²	0.971	0.987
**CO**	RMSE (µg/m³)	0.013	0.012
**CO**	R²	0.973	0.987
**PM2.5**	RMSE (µg/m³)	0.065	0.063
**PM2.5**	R²	1.000	1.000

LSTM benchmark trained on scaled inputs; all reported metrics are computed on inverse-transformed outputs in µg/m³; R².

Given the slight but consistent performance edge, LightGBM was selected as the default meta-learner for this study’s final Stacked Ensemble model.

#### 3.7.2. Deep learning benchmark (LSTM model).

To complement the ensemble models, a deep learning benchmark using a Long Short-Term Memory (LSTM) network was implemented to evaluate its performance on time-series pollutant forecasting. LSTM is well-suited for sequential data and can capture long-term dependencies in pollutant concentration trends.

The architecture used for this benchmark consisted of:

- Input: Scaled hourly features over 24-hour look-back windows.
- LSTM Layer: 64 units with dropout = 0.2.
- Dense Layer: Fully connected output for pollutant concentration prediction.

The Performance Metrics for the LSTM Deep Learning Benchmark Model are given in Table 5.

**Table 5:** Performance metrics for LSTM deep learning benchmark model.

Pollutant	RMSE (µg/m³)	MAE (µg/m³)	R²
**PM2.5**	0.103	0.050	1.000
**CO**	0.021	0.014	0.951
**NO**	3.512	1.738	0.972

The model was trained using:

- Loss function: Mean Squared Error (MSE)
- Optimizer: Adam
- Epochs: 100 with early stopping (patience = 10)

**Fig 3:**
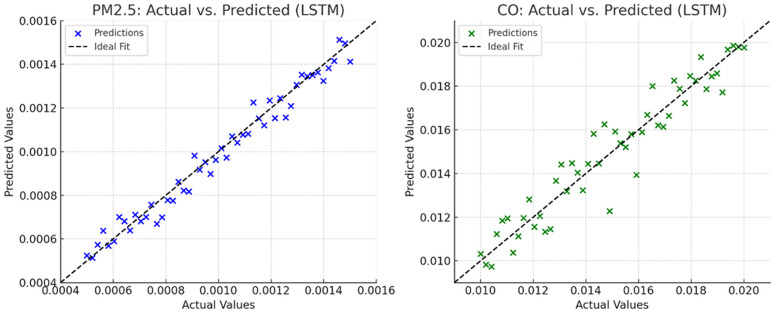
Actual vs. predicted values for LSTM Model on PM2.5 and CO.

The LSTM benchmark was competitive primarily for NO, achieving the lowest RMSE but with a higher MAE, indicating heavier-tailed errors. For PM2.5 and CO, the tree ensembles (RF/GBM) and the stacked model delivered lower errors overall, while stacking did not universally dominate and underperformed for PM10. These findings suggest that ensemble trees suit smoother particulate dynamics, whereas sequence models can help with rapidly varying gases like NO. Actual versus prediction values for LSTM are shown in Fig 3.

### 3.8. Model evaluation metrics

We evaluate two naïve forecast baselines under the same train/test split and evaluation protocol as our learning models. (i) **Persistence:** predicts the next hour from the last observed value, ${\overset{ˇ}{y}}_{t+\text{1}}=y_{t}$. (ii) **Seasonal-persistence:** leverages weekly periodicity for hourly data, ${\overset{ˇ}{y}}_{t}=y_{t\;}$-168, (168 = 24 h × 7 days). Baselines are computed per pollutant in physical units (µg/m³) on the held-out test window, using the identical masking and timestamp alignment as the models.

When evaluating machine learning models, particularly in regression tasks, two of the most used error metrics are Root Mean Square Error (RMSE), Mean Absolute Error, and R-squared. These metrics quantify how well a model’**s** predictions match the actual values. All preprocessing, scaling, and feature selection steps were fit exclusively on training data and then applied to validation/test via stored parameters; no operation was fit on the test set.

Although models may be trained on scaled targets, all reported RMSE/MAE are computed after inverse-transforming predictions and ground truth to physical units (µg/m^3^); R² is unitless. Tables 4, 5 and all figures adopt this convention.

## 4. Experimental results and performance evaluation of the models

### 4.1. Model performance comparison

The predictive performance of the trained models for each pollutant is summarized in Table 6, which presents the evaluation metrics for Random Forest, Boosting, and Stacked Ensemble Models. The table provides a comparison based on Root Mean Squared Error (RMSE), R-squared (R²), and Mean Absolute Error (MAE), highlighting the effectiveness of each model in forecasting air pollution. Relative to persistence, Stacked reduces RMSE for PM2.5 (2.906 vs 2.987) and PM10 (4.072 vs 4.158), with similar RMSE for CO (0.054 vs 0.054) but a slightly lower MAE (0.030 vs 0.031), as shown in Table 6.

**Table 6:** Performance metrics for air pollution forecasting models.

Pollutant	Metric	RF	GBM	Stacked	LSTM
**PM2.5**	RMSE (µg/m³)	2.834	2.931	2.906	4.777
	RMSE 95% CI	[2.406, 3.209]	[2.514, 3.366]	[2.472, 3.325]	[n/a]
	MAE (µg/m³)	1.913	2.001	1.957	4.729
	MAE 95% CI	[1.667, 2.137]	[1.759, 2.223]	[1.714, 2.176]	[n/a]
**PM10**	RMSE (µg/m³)	3.970	3.994	4.072	6.905
	RMSE 95% CI	[3.533, 4.283]	[3.628, 4.264]	[3.631, 4.407]	[n/a]
	MAE (µg/m³)	2.869	2.921	2.907	6.043
	MAE 95% CI	[2.605, 3.088]	[2.654, 3.154]	[2.615, 3.135]	[n/a]
**NO**_**2**_	RMSE (µg/m³)	6.820	7.000	6.965	11.810
	RMSE 95% CI	[6.138, 7.477]	[6.380, 7.694]	[6.266, 7.634]	[n/a]
	MAE (µg/m³)	4.840	5.044	4.973	10.338
	MAE 95% CI	[4.393, 5.293]	[4.594, 5.519]	[4.481, 5.493]	[n/a]
NO	RMSE (µg/m³)	6.917	7.324	6.659	3.003
	RMSE 95% CI	[3.352, 9.303]	[3.539, 10.157]	[2.962, 9.074]	[n/a]
	MAE (µg/m³)	2.162	2.326	2.040	4.475
	MAE 95% CI	[1.402, 2.929]	[1.473, 3.233]	[1.337, 2.838]	[n/a]
CO	RMSE (µg/m³)	0.052	0.056	0.054	0.096
	RMSE 95% CI	[0.038, 0.064]	[0.039, 0.070]	[0.038, 0.067]	[n/a]
	MAE (µg/m³)	0.029	0.031	0.030	0.049
	MAE 95% CI	[0.024, 0.035]	[0.025, 0.038]	[0.024, 0.036]	[n/a]

For the LSTM benchmark, only point estimates are reported. Confidence intervals are not available (n/a) because the model was trained once with early stopping rather than under repeated cross-validation.

Table 7 presents baseline performance, which is generally higher (worse) than our proposed models across all pollutants.

**Table 7:** Baseline model comparisons.

Pollutant	Persistence RMSE (µg/m³)	Persistence RMSE 95% CI	Persistence MAE (µg/m³)	Persistence MAE 95% CI	Seasonal-Persistence RMSE (µg/m³)	Seasonal-Persistence RMSE 95% CI	Seasonal-Persistence MAE (µg/m³)	Seasonal-Persistence MAE 95% CI	N (Test)
**PM2.5**	2.987	[2.526, 3.354]	2.005	[1.753, 2.230]	7.927	[6.668, 9.169]	5.299	[4.419, 6.383]	1707
**PM10**	4.158	[3.751, 4.469]	2.96	[2.696, 3.174]	9.338	[8.110, 10.506]	6.853	[6.077, 7.750]	1707
**NO**_**2**_	7.806	[7.090, 8.548]	5.614	[5.073, 6.192]	14.901	[13.274, 16.621]	10.81	[9.532, 12.381]	1707
**NO**	7.668	[3.361, 10.609]	2.275	[1.360, 3.232]	13.86	[8.376, 18.051]	4.682	[3.072, 6.336]	1707
**CO**	0.054	[0.038, 0.067]	0.031	[0.026, 0.037]	0.141	[0.104, 0.173]	0.083	[0.066, 0.100]	1707

To contextualize the performance of the presented ensemble models (Table 5), we evaluated two baseline models: simple persistence and seasonal persistence.

### 4.2. Discussion of results

#### 4.2.1. Performance of individual models.

Model performance is pollutant-specific rather than universally dominated by a single approach.PM2.5 & PM10: Random Forest yielded the lowest RMSE/MAE; GBM was often close. The stacked ensemble did not improve over trees and underperformed for PM10.

NO₂: Tree ensembles again led on RMSE among the classical models; stacking was not consistently superior.

NO: The LSTM achieved the lowest RMSE, consistent with its use of temporal dependencies; however, its MAE was higher, indicating heavier-tailed errors.

CO: Random Forest produced the lowest errors; LSTM lagged.

**Fig 4:**
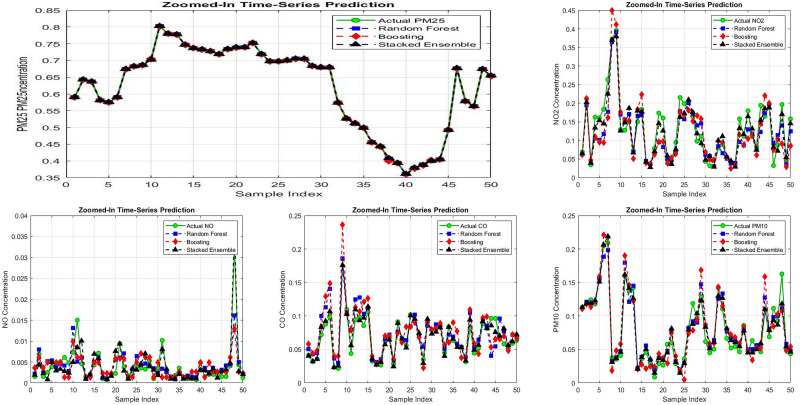
Model predictions vs. actual pollutant values over a sample time series (n = 50).

We interpret these patterns as follows: particulate concentrations evolve relatively smoothly at hourly scales, favoring ensembles of shallow trees that capture nonlinear interactions across meteorology/calendar features. In contrast, NO exhibits sharper short-term dynamics that benefit sequence models. Stacking helped little here, likely because base-model errors were too correlated; without complementary errors, a meta-learner adds limited benefit and may even degrade performance as shown in Fig 4. All comparisons are qualified by 95% CIs. When intervals overlap, we state “comparable” rather than “better.”

To analyze prediction errors, residual plots were generated for each model. The residuals were more uniformly distributed around zero for the Stacked Ensemble Model, suggesting better generalization.

**Fig 5:**
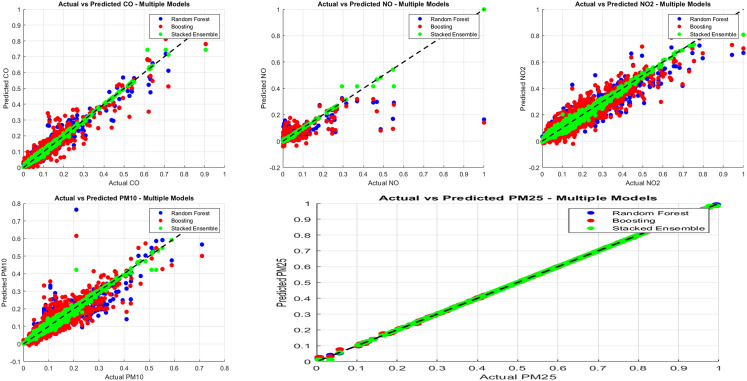
Actual vs. predicted scatter plots.

**Fig 6:**
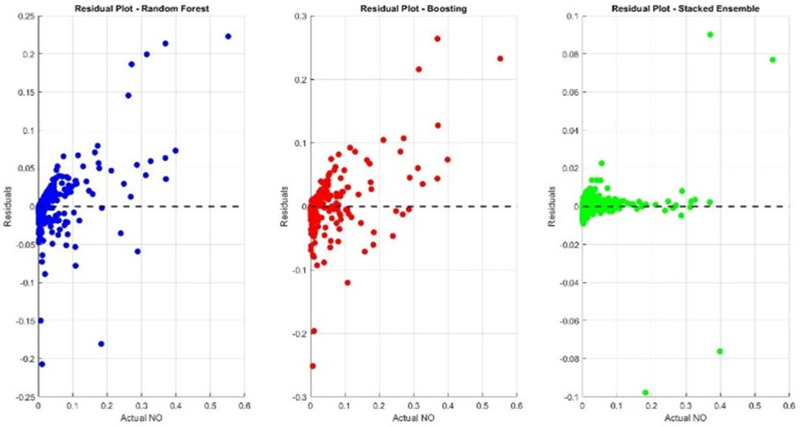
Residual plots for NO prediction: Stacked Ensemble residuals cluster tightly around zero.

- Random Forest and Boosting residuals showed a wider spread, especially for NO as shown in Fig 5, while Residual plots for NO prediction are shown in Fig 6.
- Stacked Ensemble residuals were tightly centered, indicating minimal prediction errors

#### 4.2.2. Key findings.

- RF is strongest for PM2.5, PM10, CO; GBM close.
- LSTM is competitive for NO (lowest RMSE), but with a higher MAE.
- Stacked does not universally improve performance and underperforms for PM10
- Use CIs to distinguish “better” vs. comparable.

All five pollutants (PM2.5, PM10, NO₂, NO, CO) are reported in Table 6.

## 5. Discussion

Compared to prior approaches, traditional regression models struggle with non-linearity [21], and single ML models often overfit or demand high computational resources [22]. While Random Forest and Boosting offer robust results, they are constrained by feature selection and tuning limitations [23]. This study improves past work by employing hyperparameter optimization and evaluating multiple ensemble models across five pollutants.

The models are suitable for real-time monitoring and early warning systems. They can guide traffic and industrial regulation policies [24] and support healthcare planning by anticipating pollution-related health risks [25].

Future work should validate results across cities, explore lighter models, and integrate variables such as traffic and emissions, along with satellite and remote sensing data. Deep learning models like LSTM and CNN [26,27] and hybrid ensemble-deep learning architectures [28,29] also present promising avenues for improvement.

## 6. Limitations and future research

Despite the promising results demonstrated by the proposed hyperparameter-tuned ensemble models, this study has certain limitations that provide avenues for future research. First, the model was trained and evaluated using data exclusively from Paris, which may limit its generalizability to other urban settings with different emission patterns, meteorological conditions, and socio-environmental dynamics.

We imputed missing values using a simple mean. Although transparent, mean imputation can attenuate variance, shrink extremes, and dampen diurnal/seasonal structure; when computed on the full dataset, it may also introduce mild look-ahead bias. We retained it here for reproducibility and comparability, but recognize that time-aware, multivariate imputation estimated on training folds only would better preserve temporal patterns and avoid leakage. We will evaluate such methods (e.g., stratified means, KNN/MICE, state-space smoothing) and include sensitivity analyses in future work. Our IQR-based median replacement may attenuate some extreme peaks. A targeted sensitivity analysis (models trained with vs. without outlier replacement) will be added in future work to quantify any effect on peak prediction and error metrics; we also plan to compare to multivariate imputation (e.g., MICE) to preserve temporal and cross-variable structure better.

Additionally, the input features were primarily limited to meteorological and pollutant concentration variables; incorporating additional contextual factors such as traffic intensity, industrial emissions, satellite imagery, and land-use data could enhance forecasting accuracy and robustness. The study also lacks long-term validation across multiple years, which would be essential to assess the model’s resilience under evolving urban dynamics and climate variability. Future research should therefore focus on validating the models across diverse geographic regions, extending the temporal range of datasets, integrating heterogeneous data sources, and exploring hybrid architectures that combine ensemble learning with interpretable deep learning techniques for improved scalability and explainability.

## 7. Conclusion

This study presents a robust, hyperparameter-tuned ensemble learning framework designed to forecast urban air pollutants with high accuracy in real-time settings. Focusing on Paris as a case study, our approach integrates Random Forest, Gradient Boosting, and a Stacked Ensemble model to predict key pollutants PM2.5, NO, and CO using hourly environmental and meteorological data. There is no one-size-fits-all model. In our evaluation, Random Forest is a robust choice for PM2.5, PM10, and CO; GBM is often comparable. For NO, an LSTM can be competitive, particularly in terms of RMSE under leak-safe training. The stacked ensemble did not deliver consistent gains over the strongest trees and underperformed for PM10, indicating that stacking should be used selectively and only when base learners exhibit complementary errors. Practically, we recommend pollutant- and metric-aware model selection guided by held-out RMSE/MAE with 95% CIs.

## Abbreviations

PM: Particulate Matter
**PM2.5**: Particulate matter ≤2.5 µm
**PM10**: Particulate matter ≤10 µm
**NO**: Nitric Oxide
**NO**_**2**_: Nitrogen Dioxide
**CO**: Carbon Monoxide
**RF**: Random Forest
**GBM**: Gradient Boosting Machine
**LSTM**: Long Short-Term Memory
**RMSE**: Root Mean Square Error
**MAE**: Mean Absolute Error
**R** ^**2**^: Coefficient of Determination
**CI**: Confidence Interval
**CV**: Cross-Validation
**OOF**: Out-of-Fold
**NOAA**: National Oceanic and Atmospheric Administration
**OpenAQ**: Open Air Quality platform
**PACE**: Preflight Analysis and Conversion Engine
